# Renal Tumors in Young Adults: Is Preoperative Computer Tomography Imaging Suggestive for the Nature of the Tumors?

**DOI:** 10.3390/diagnostics10060380

**Published:** 2020-06-07

**Authors:** Andreea Zaharie, Sorana D. Bolboacă, Tudor Moisoiu, Dan Burghelea, Gheorghita Iacob, Liviu Ghervan, Florin Ioan Elec

**Affiliations:** 1 Department of Medical Imaging, “Leon Daniello” Pulmonology Hospital, Cluj-Napoca, Bogdan Petriceicu Hașdeu Street, no. 6, 400332 Cluj-Napoca, Romania; 2 Department of Medical Informatics and Biostatistics, Iuliu Hațieganu University of Medicine and Pharmacy Cluj-Napoca, Louis Pasteur Str., no. 6, 400349 Cluj-Napoca, Romania; 3 Department of Urology, Clinical Institute of Urology and Renal Transplantation, Clinicilor Str., no. 4–6, 400006 Cluj-Napoca, Romania; tmoisoiu@gmail.com (T.M.); dr.danburghelea@gmail.com (D.B.); liviughervan@yahoo.com (L.G.); 4 Department of Pathology, Clinical Institute of Urology and Renal Transplantation, Clinicilor Str., no. 4–6, 400006 Cluj-Napoca, Romania; iacob.gheorghita@gmail.com; 5 Department of Urology, Iuliu Hațieganu University of Medicine and Pharmacy Cluj-Napoca, Clinicilor Str., no. 4–6, 400006 Cluj-Napoca, Romania

**Keywords:** computer tomography (CT), kidney tumors, renal cell carcinoma

## Abstract

Renal cell carcinoma (RCC) accounts for 2–3% of all adult malignant neoplasms and is even rarer in patients under 45 years old. Clear-cell carcinoma represents most of the pathological subtypes. Our study aimed to investigate the association between preoperative computer tomography imagistic evaluation and histopathological diagnosis of renal tumors in young adults. Patients younger than 45 years old with renal tumors who were referred for medical treatment at the Clinical Institute of Urology and Renal Transplantation Cluj-Napoca from 2012 to 2019 were considered eligible for the study. Medical charts were retrospectively reviewed, and patients with complete data regarding preoperative diagnostic, histopathological evaluation, and follow-up data, regardless of gender, were included in the study. Sixteen patients younger than 45 years fulfilled all the inclusion criteria and were evaluated. With two exceptions, the evaluated patients were in a T1 and T2 stage, with no vascular invasion or of the adjacent organs. Two-thirds of our patients had a clear-cell renal cell carcinoma. None of our patients fitted in the low complexity surgery category of the R.E.N.A.L. Nephrometry Score and 37.5% of them benefited from partial nephrectomy. Half of the suppositions made based on imaging were concordant with the histopathology report. Fifteen of the patients showed no recurrence during the respective follow-up interval. Computer tomography imaging reports showed on our sample a higher concordance with the histopathological report in the more common subtypes (namely Renal Clear Cell RCC), with typical appearances.

## 1. Introduction

Kidney and renal pelvis malignancies account for 4.2% of all new cancer cases discovered in 2019 [1] and are even rarer in patients under 45 years old [2]. Renal cell carcinoma (RCC) accounts for 90% of all kidney tumors [3], and clear-cell carcinoma represents over 75% of the pathological subtypes (generally identified at a more advanced stage and with poor prognosis [4]), followed by papillary and chromophobe subtypes [5].

The incidental detection of renal masses [6,7] is a consequence of the widespread use of thoracoabdominal imaging (CT—Computed Tomography or MRI—Magnetic Resonance Imaging) ordered for non-urological related complains [8]. Renal tumors are nowadays more frequently diagnosed in patients compared to 1958–1969 [9], and the rate of incidentally diagnosed renal cell carcinoma (RCC) increased from 7% in 1971 [10] to 21.1% in 1978–1987 [11] and 57% in 2001–2010 [8].

In the younger group, all the alternative diagnoses have to be taken into consideration when a renal tumor is identified.

Radiological instruments are mandatory in characterizing, staging, surgical planning, and follow-up. The CT (computer tomography) diagnosis algorithm for renal tumors (unenhanced and three phases post-contrast) in symptomatic patients is proposed and used in clinical practice [12]. The Disease Focused Panel on Renal Cell Carcinoma of the Society of Abdominal Radiology recommends for pre-partial nephrectomy patients: arterial phase, nephrographic, and excretory phases, with coronal and sagittal reconstructions. The CT with portal venous phase, with an optional late arterial phase surveillance, should be carried out for patients treated with radical nephrectomy or systemic therapy in patients with a high risk of metastases to the liver or pancreas. Computed-tomography scans are usually readily available, have a reduced cost compared to MRI, and are a good solution for patients with claustrophobia since the scan time is shorter. On the other hand, CT scans use ionizing radiation (of particular concern in young patients), have a lower sensitivity in low BMI patients (because of the low soft-tissue contrast), and require the use of contrast injection (which might be problematic in patients with altered kidney function). Unfortunately, sometimes a CT scan is not enough, and an MRI must be performed to discern specific characteristics of the tumor (Table 1, [13]).

The MRI evaluation could be useful when a papillary RCC is suspected, or when the assessment of the intracellular fat or the septa is of interest [14]. Gray and Harris [15] provide algorithms of diagnosis and management for RCC and also describe imaging evaluation of incidentally discovered renal masses.

Even though the peak age for RCC onset is in the seventh decade of life, in 3-7% of cases, RCC is also seen in young adults (<40 years) [16,17,18]. Von Hippel-Lindau, Birt-Hogg-Dube, tuberous sclerosis, hereditary papillary RCC, hereditary leiomyomatosis and RCC, succinate dehydrogenase kidney cancer, Cowden syndrome, microphthalmia-associated transcription factor, paraganglioma syndromes, familial non-syndromic clear cell RCC, and hyperparathyroidism jaw tumor are RCC syndromes described in the scientific literature along with associated tumor histology [19]. Partial nephrectomy should be attempted whenever feasible from an oncological standpoint and whenever technically possible [20]. Aslan et al. [21] reported clear cell RCC as the more frequent type of RCC in young adults (22%), with benign masses more common in women (35% vs. 13.6%), and a four years survival of 93.8%. Abou et al. [22] reported an incidental finding, with RCC in young adults, of 40% that were less likely to develop metastases and had better survival compared to symptomatic patients (*p* = 0.001). Sanchez-Ortiz et al. showed that young adults compared to older adults had more frequent unfavorable histological features such as sarcomatoid, rhabdoid, unclassified, medullary or collecting duct (23.6% vs. 1.7%, *p*-value = 0.02), a higher incidence of lymph node metastases at the presentation (25% vs. 15%, *p*-value < 0.03), and a better disease-specific and recurrence-free survival [23].

Our study aimed to investigate the association between preoperative computer tomography imagistic evaluation and histopathological diagnostic of renal tumors in young adults.

## 2. Materials and Methods

The study was conducted in a single clinical regional center, namely the Clinical Institute of Urology and Renal Transplantation in Cluj-Napoca. The Ethical Committee of the Clinical Institute of Urology and Renal Transplantation approved the study (approval no. 2 from 01/02/2020) with exempt informed consent since only recorded data in medical charts were used. All patients younger than 45 years old with a renal mass, who were operated on in the last eight years (from 2012 to 2019), were eligible for this study. We recorded demographic data of the patients, TNM (Tumour, Node, Metastasis) classification, the complexity of the tumor R.E.N.A.L. Nephrometry Score [24], computer tomography (CT) data, type of surgery, histopathological examination findings, outcome, and follow-up data. The patients were evaluated clinically and with a CT at six months in the first two years and yearly afterward.

The R.E.N.A.L. Nephrometry Score (NS) was introduced to quantify the characteristics of renal masses on cross-sectional imaging, to allow standardized reporting of kidney masses, assist with objective discussions of risks with patients, and contribute to surgical decision making. A NS of 4 to 6 means a low complexity of the tumor with a 6.4% potential complication rate, and a NS of 7 to 9 suggests an intermediate complexity with an 11.1% risk of developing a postoperative complication. A NS over 10 indicates a high complexity, and patients are usually more likely to undergo radical nephrectomy [25].

The NS was calculated to assess the complexity for surgical decision-making, using the largest diameter of the tumor, the grade of protrusion of the tumor (% mass exophytic/endophytic), nearness to collecting system or sinus, location relative to polar lines, proximity to the renal artery and the renal vein (Table 2).

**Figure 1:**
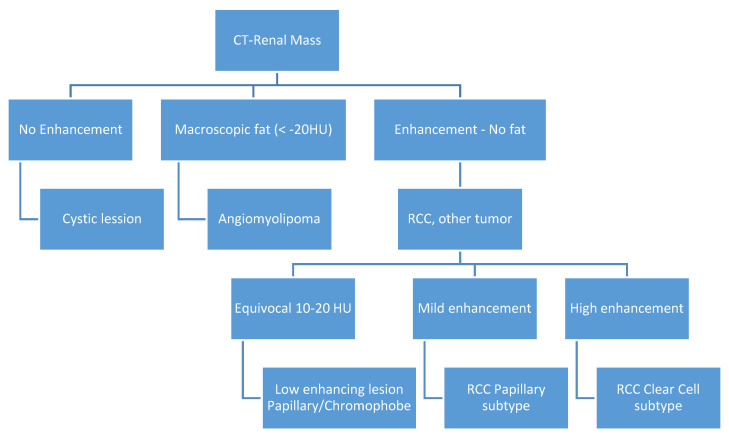
Computer tomography (CT) algorithm for renal mass classification (*HU—Hounsfield Units*).

The CT scans of the patients were performed in outside medical practices, most of them for non-specific symptoms or other indications, thus the protocols were not standardized. Nevertheless, all of them had an arterial and venous phase, some of them had a non-enhanced phase performed, and some of them also had excretory phases performed, but neither were included in our article due to the inconsistency among cases. One experienced radiologist blinded to the diagnosis retrospectively assessed the CT scan examinations and reported the type of tumor according to the imaging tumor characteristics summarized in Figure 1.

Accurate histologic characterization of RCC is essential from prognostic and management perspectives and was done accordingly to WHO Classification of Tumors of the Urinary System and Male Genital Organs (2016) [26]. One experienced pathologist assessed all cases in a clinically blinded evaluation setting.

### Data Analysis

Data were summarised with descriptive metrics using absolute and relative frequencies. Concordance between preoperative CT imaging and histological analysis was achieved when both methods gave the same diagnosis. The concordance was reported as Yes/No when the preoperative CT diagnosis contained a list of possibilities and one from the list was confirmed by histological diagnostic.

## 3. Results

Even though 370 patients with renal tumors were considered over a period of 8 years, only 43 were under 45 years. Sixteen patients aged less than 45 years old (with preoperative CT imaging, histopathology report, and follow-up data) with surgically treated renal masses were included in this study. With one exception, the patients had no apparent symptoms, and the kidney masses were incidentally identified, either during a routine ultrasound or with the occasion of an investigation for a different benign pathology, which followed the urological consultation or further investigations. The exception was a 37 years old male patient with left lumbar pain. Most patients’ tumors were in a T1 stage, one-third of the patients were in stage T2, and only 12.5% were in a T3 stage (Table 3). The patients’ clinical and radiological findings are summarized in Table 3.

**Figure 2:**
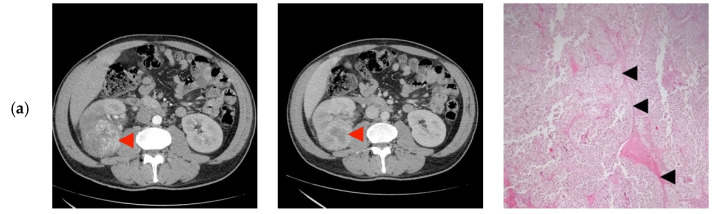

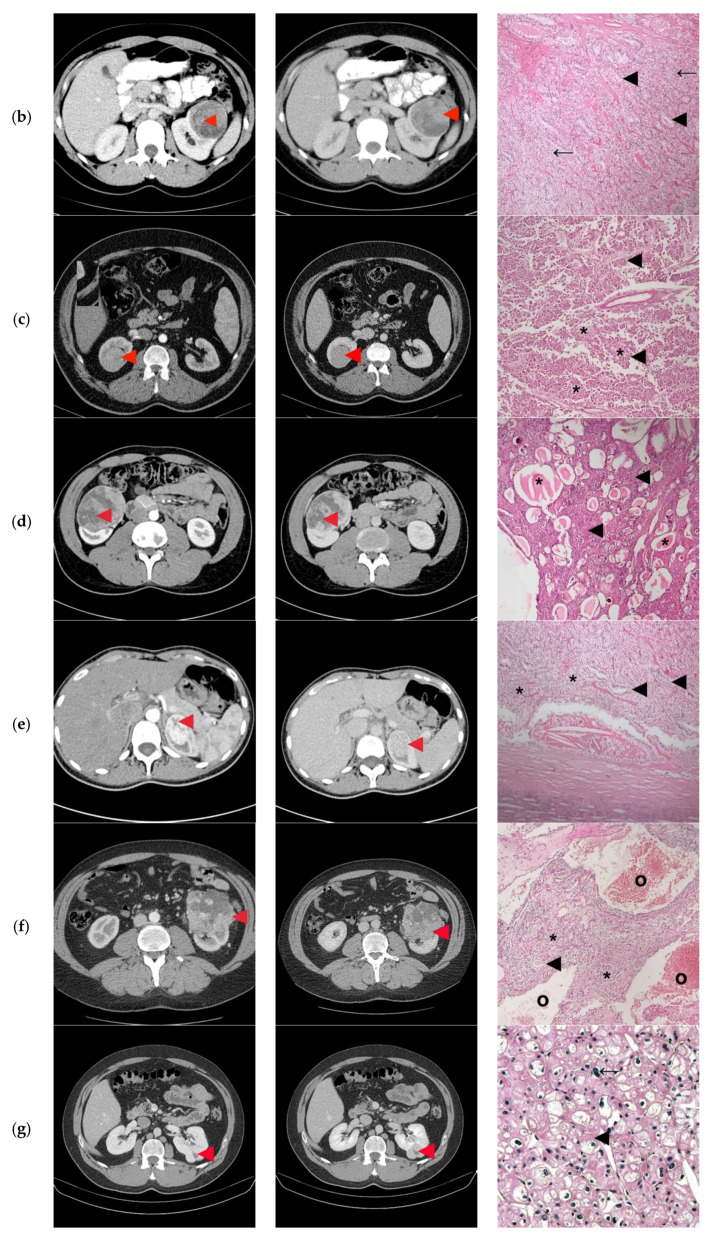

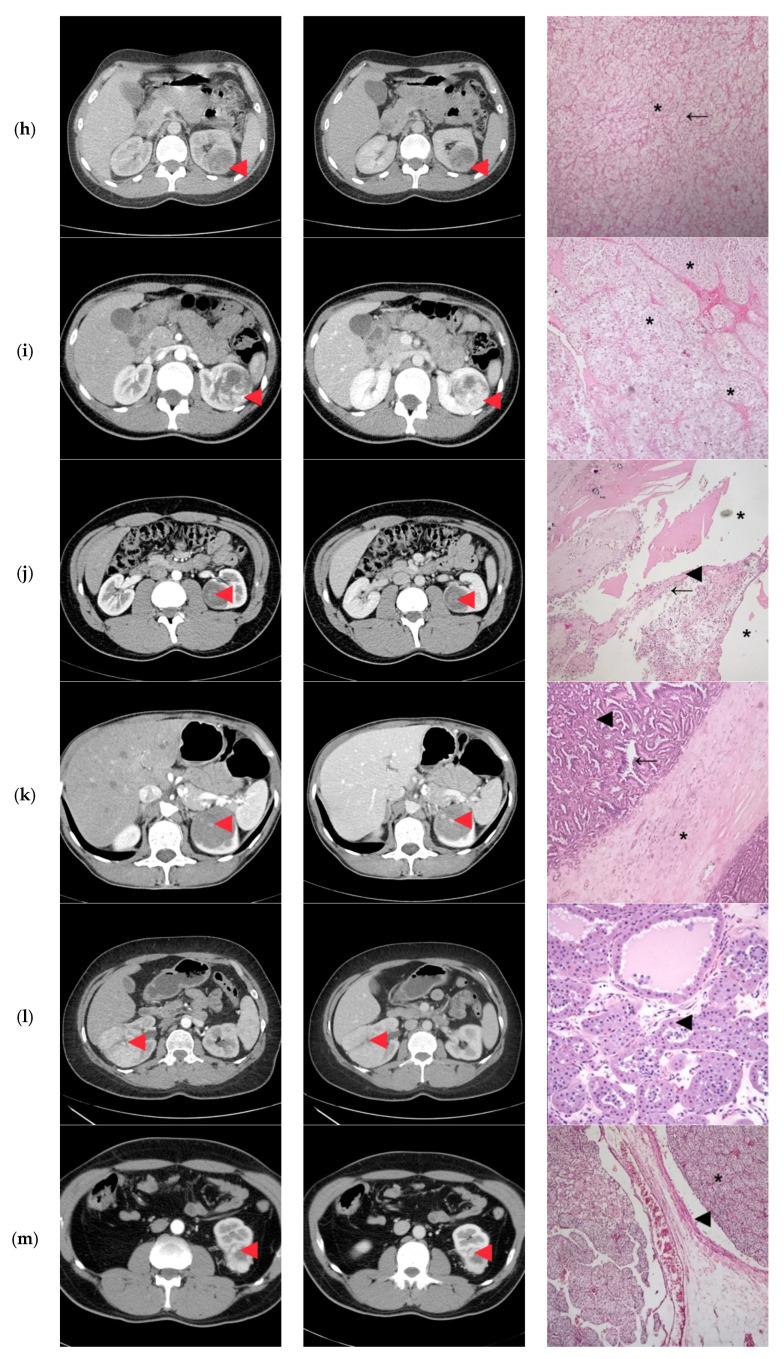

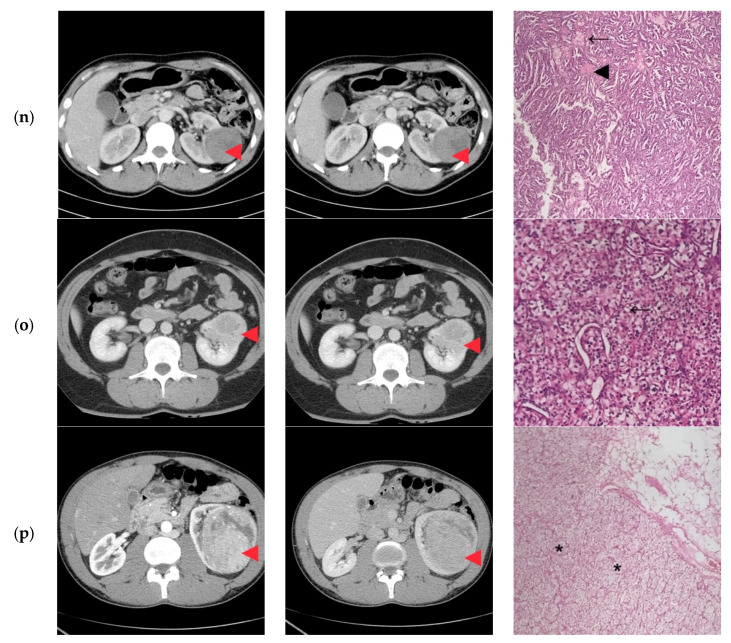
Computer tomography findings in the cortico-medullary and nephrographic phases; Histopathological examination (**a**) Heterogeneous peripheral contrast enhancement, like the renal cortex (◄); Clear Cell RCC, Fuhrman grade 2. Tumor with compact and alveolar architecture (◄) of cells with clear cytoplasm, distinct cell boundaries and nuclei with nucleoli prominent at 100×. (**b**) Moderate nodular enhancement (◄); Clear Cell RCC, Fuhrman grade 1. Tumor with alveolar (←) and tubular architecture (◄) of cells with clear cytoplasm, distinct cell boundaries and nuclei with inconspicuous nucleoli at 100×. (**c**) Faint intra-tumoral contrast enhancement with mild progression in the nephrographic phase (◄); Papillary-RCC, type II. Tumor with papillary architecture (*) with pseudostratified layers (◄) of large cells with abundant eosinophilic cytoplasm, atypical nuclei with prominent nucleoli at 100×. (**d**) Heterogeneous contrast enhancement, less than the renal cortex in both phases, with an important necrotic component (◄); Adult cystic nephroma. A tumor composed of various sized cysts (◄) lined by flat cells separated by fibrous septa that contain smooth muscle (*) at 100×. (**e**) Intense contrast in the cortico-medullary phase, with an apparent central “scar” (◄) and less contrast enhancement compared to the renal cortex in the nephrographic phase; Clear Cell RCC, Fuhrman grade 1. Tumor with alveolar (*) and tubular (◄) architecture of cells with clear cytoplasm, distinct cell boundaries, and nuclei with inconspicuous nucleoli at 100×. (**f**) Bulky exophytic tumor with nodular peripheral contrast enhancement, like the renal cortex on the cortico-medullary phase and less than this on the nephrographic phase (◄); Clear Cell RCC, Fuhrman grade 2. Tumor with alveolar (*), tubular (◄) and cystic (o) architecture of cells with clear cytoplasm, distinct cell boundaries, and nuclei with nucleoli prominent at 100×. (**g**) Peripheral contrast enhancement like the renal cortex with an apparent “central scar” (◄) on the cortico-medullary phase and wash-out on the nephrographic phase, with the persistence of the “central scar”; Multicentric Chromophobe-RCC. Flocculent cytoplasm that condenses around the edges (“plant cell-like”, ◄) and pleomorphic nuclei (←) at 400×. (**h**) Faint peripheral enhancement on both phases (◄); Clear Cell RCC, Fuhrman grade 1. Tumor with the alveolar architecture of cells with clear cytoplasm (*), distinct cell boundaries (←), and nuclei with inconspicuous nucleoli at 100×. (**i**) Bulky tumor with peripheral enhancement (◄), like the renal cortex in the cortico-medullary phase, with progressive filling in the nephrographic phase; Clear Cell RCC, Fuhrman grade 2. Tumor with alveolar architecture (*) of cells with clear cytoplasm, distinct cell boundaries and nuclei with nucleoli prominent at 100×. (**j**) Faint spotted intra-tumoral enhancement on both phases (◄); Multiloculated cystic RCC, Fuhrman grade 1. Tumor with cystic architecture (*), with thin fibrous septae (◄) lined by clear cells (←). Tumor cells with inconspicuous nucleoli at 100×. (**k**) Faint, spotted intra-tumoral enhancement on both phases (◄); Metanephric adenofibroma. A tumor composed mainly of tightly packed tubules (◄) and secondarily long branching and angulated ducts (←). Tumor cells have scant cytoplasm and nuclei are small with no nucleoli, with no mitotic figures. Stroma is present as significant fibrous septa (*) at 100×. (**l**) Peripheral enhancement is like the renal cortex with the presence of a central non-enhancing area (“central scar”) (◄), on both phases; Oncocytoma. Small oncocytic cells (◄) with round, regular nuclei, no mitosis at 200×. (**m**) Peripheral enhancement like the renal cortex on the cortico-medullary phase and discretely less than the renal cortex on the nephrographic phase (◄); Clear Cell RCC, Fuhrman grade 1. Tumor with alveolar architecture (*) of cells with clear cytoplasm, distinct cell boundaries, and nuclei with inconspicuous nucleoli. The tumor invades a segmental renal vein (◄) at 100×. (**n**) Discrete nodular, peripheral enhancement (◄), less than the renal cortex on both phases; Papillary RCC, type I. Tumor composed of small cuboidal cells, with scant cytoplasm and round nuclei without nucleoli, arranged on a single layer on papillary cores (◄). Some of the papillary cores contain foamy macrophages (←) at 100×. (**o**) Peripheral enhancement less than the renal cortex on both phases (◄); Clear Cell RCC, Fuhrman grade 1. Tumor with a compact and alveolar architecture of cells with clear cytoplasm (←), distinct cell boundaries, and nuclei with inconspicuous nucleoli at 100×. (**p**) Bulky tumor with peripheral enhancement, less than the renal cortex on both phases (◄), with wash-out in the nephrographic phase; Clear Cell RCC, Fuhrman grade 1. Tumor with alveolar architecture (*) of cells with clear cytoplasm, distinct cell boundaries, and nuclei with inconspicuous nucleoli at 100×.

None of the patients had tumor invasion of the renal vein or the adjacent organs, calcifications, or macroscopic fat (Figure 2).

None of our patients fitted into the low complexity surgery category of the NS. In 56.25% of patients, the NS was compatible with a high complexity surgery, while 43.75% were classified as intermediate complexity. Consequently, six patients had a partial nephrectomy, while the rest had a radical nephrectomy.

Clear cell carcinoma was proved histologically in two-thirds of patients (62.5 %), with only two patients with papillary-RCC and one with chromophobe-RCC (Table 4). Half of the CT imaging diagnoses were concordant with the pathology report (Table 4).

The follow-up ranged from less than one year to seven years, with a median of two years (interquartile range from 1.5 to 3 years). Fifteen of the patients had no recurrence during the follow-up. One patient presented with a solitary diaphragmatic metastasis at two years, treated surgically and complementary with Sunitinib, with no consequent recurrence at five years. Our patient with multicentric chromophobe-RCC was genetically tested for the FLCN (folliculin) gene mutation with negative results.

## 4. Discussion

Only 8/16 of the preoperative CT diagnoses were concordant with the pathology report, and in 11/16 cases the CT diagnoses overlapped with the histological diagnosis. Few unexpected and striking discordances were observed in our study. For instance, the CT images of the patient diagnosed with multiloculated cystic clear-cell RCC Furhman 1 were not indicative of this histology. Usually, this type of tumor is visible as a cystic lesion classified as at least Bosniak IIF [27]. Furthermore, the patient diagnosed with multicentric chromophobe RCC had CT images that showed only one tumor that resembled that of an oncocytoma, with a central, non-enhancing scar.

On the other hand, only one of the patients diagnosed with papillary RCC had this differential included in the final report. These types of tumors are generally hypo-vascular compared to the renal parenchyma with progressive uptake in the nephrographic phase [27]. Also, our case series includes a rather typical appearance of an oncocytoma, with a central non-enhancing scar.

The highest degree of concordance was in the cases of clear cell RCC, the most frequent histology, and thus with well-known imaging characteristics. As expected, the radiologists did not predict the rarer histologies like metanephric adenomyofbroma, neuroblastoma, and Chromophobe-RCC (Table 4).

Most of our patients had no symptoms, the renal masses being incidentally identified. However, the percentage of incidental identification of renal mass is higher in our study as compared to King et al., who reported a value of 60% [20]. Half of the investigated patients were younger than 35 years, with slightly more men than women (9 vs. 7; Table 3), the result similar to that of Eggener et al. [28].

Even in the younger population group, renal cell carcinoma dominates the histological results, our patients’ results corresponding to this entity in a proportion of 62.5%. Across all age groups, renal cell carcinoma is discovered in a metastatic stage in about one-third of patients. Fortunately, in young patients, the tumors seem to be at a lower stage and a lower grade [29], thus making their management less debilitating. Even when more aggressive therapy is required, younger patients seem to have a better response, perhaps due to their increased performance score. More than half of our patients had a tumor staged T1, and a minority of them (12.5%) were staged T3 (Table 4). Also, we had only one patient with a solitary diaphragmatic metastasis treated surgically and with chemotherapy with a recurrence-free period of 5 years. None of the patients had a vascular invasion or invasion at adjacent organs.

Renal tumors in young patients are somehow surprising, considering that other solid organ cancers such as breast, colorectal, and prostate carry a more discouraging prognosis [12]. In contrast, renal tumors have been proved to show better survival and a more extended progression-free period than their older counterparts [3,4,5,6,12,18,23,29]. A similar progression-free period was also observed in our sample, with the majority of patients being disease-free during the follow-up period.

Nevertheless, several studies reinforce the same prognostic factors for renal cell carcinoma: pTNM tumor stage, Fuhrman nuclear grade, circumstances of discovery, and tumor size. As far as tumor size is concerned, Frank et al. [30] demonstrated a high percentage of malignant histology associated with the bigger tumors. In the case of our patients, one of the two patients pathologically classified T3 had a clear-cell RCC Fuhrman grade 3, while out of five patients classified T2, two of them had a Clear Cell RCC, Fuhrman grade 2 (Table 4).

Partial nephrectomy is the surgical treatment of choice. It is recommended whenever technically possible because it is associated with a longer life expectancy for this category of patient, but also due to the risk of possible metachronous involvement of the contralateral kidney [20]. All the patients included in our case series had a RENAL score that included them in intermediate and high complexity surgery, with a slight majority favoring the latter. Even though the preferred course of treatment for young people, for whom we expect a longer survival, is partial nephrectomy, only one-third of our patients benefited from this surgical approach (Table 4). The individual decisions were mainly guided by the R.E.N.A.L. Nephrometry Score, the surgeon’s confidence, and the patients’ preference.

In general, whenever possible, young patients diagnosed with renal tumors should benefit from genetic counseling and testing (Chromophobe-RCC is associated with Birt-Hogg-Dubé syndrome, 10% of clear-cell RCC harbor Xp11.2/TFE3 translocation) [20]. In our case series, we had one patient with multicentric Chromophobe-RCC, and the suspicion of Birt-Hogg-Dubé syndrome was raised. The patient had a negative result for the FLCN gene mutation. None of our patients benefited from MRI scans since the waiting time is usually very long because of the lack of availability, and the patients need a prompt, timely, and accurate diagnosis. Abdominal and pelvic MRI and chest CT are recommended for the surveillance of possible local or distant recurrences considering their young age, long survival, and the extended follow-up. Age is an independent prognostic factor associated with a better prognosis and longer progression-free period in the case of kidney tumors, unlike other tumors in younger patients [29].

The limitations of our study consist of a small cohort of patients due to the rarity of this pathology among young patients. On the other hand, the CT scans that incidentally identified the renal masses were not performed in our university clinic. Thus, not all the recommended CT phases were performed, and for the benefit of the patients’ care we decided not to repeat the CT examination. Furthermore, this was a retrospective study, relying on medical charts, as they were completed when the patient was under medical observation. Thus, neither the precision of the data not the availability of information could be controlled and the evaluated sample of patients was small.

A young adult with a kidney tumor should be treated under the assumption of the worst-case scenario regardless of the computer tomography appearance. Consequently, the entire spectrum of renal tumors should be considered keeping in mind that women are more likely to have benign tumors [16,28], while oncocytoma is relatively infrequent in young patients [17]. Clear Cell RCC was the most frequent kidney tumor in young patients and was discovered in an earlier stage with better survival after the applied treatment. Even if the results obtained on such small samples as our have limited power, these results come to show that, although CT imaging plays an essential role in the identification, evaluation of the extent of the disease, and the follow-up, it cannot always pinpoint the true nature of a renal tumor.

## 5. Conclusions

Renal tumors are infrequent below 45 years, but when they occur a wider range of diagnoses should be considered, even though Clear Cell RCC was the most commonly seen in our sample.

Computer tomography reports showed a higher concordance with the histopathological report in case of common subtypes, with typical appearances. Even though imaging studies cannot always reveal the tumors’ nature, they are recommended to be used both for diagnosing a tumor and its extent, as well as for postoperative surveillance. Clinically, whenever a kidney tumor is discovered in a young patient, the worst-case scenario should be considered and acted upon accordingly.

## Figures and Tables

**Table 1:** Imaging recommendation of incidental renal masses (adapted from [13]).

Renal Masses	What?	Additional Imaging?
Incomplete characterized incidental renal masses at unenhanced CT		
Any size, homogenous low attenuation (≤20 HU) <1 cm/smaller than 2× section thickness, attenuation >20 HU Homogenous hyperattenuating (≥70 HU)	Probably benign cyst	Probably, not necessarily
With fat (<10 HU) and calcifications Attenuation >20 HU or <70 HU in any part Heterogeneous with septa, wall thickening, mural nodules, or calcification	Possible malignant	Maybe warranted *
Incomplete characterized incidental renal masses at contrast-enhanced CT		
<1 cm/smaller than 2× section thickness with homogenous low attenuation ≤20 HU or >20 HU due to volume averaging	Probably benign cyst	Probably, not necessarily
Heterogeneous and without fat (<10 HU) With fat (<10 HU) and calcifications Attenuation >20 HU in any part not due to partial volume averaging	Possible malignant	Maybe warranted *

* masses < 1 cm: additional imaging after 3 to 6 months.

**Table 2:** R.E.N.A.L. Nephrometry Score calculation (adapted from [24]).

RENAL	1 pt	2 pts	3 pts
Radius diameter (cm)	≤4	>4 but <7	≥7
Exophytic/Endophytic	≥50% Exophytic	<50% Endophytic	Entirely Endophytic
Nearest sinus or collecting system (mm)	≥7	>4 but <7	≤4
Anterior/Posterior	Nonnumerical suffix a, p, x, h		
Location, Polar lines	Entirely above or below the polar lines	The lesion crosses the polar lines	>50% of the mass crosses the polar line, or the mass is located entirely between the polar lines

**Table 3:** Characteristics of patients, renal masses, and computer tomography diagnosis.

ID	Age (years)/Gender	TNM	Tumor Diameter (mm)	R.E.N.A.L. Nephrometry Score	Preoperative CT Imaging Diagnosis
1	45/M	cT2N0	73	9	Clear Cell RCC
2	27/F	cT1bN0	60	8	Clear Cell RCC
3	38/M	cT1bN0	47	10	Papillary/Clear Cell RCC Oncocytoma
4	24/M	cT2N0	85	10	Clear Cell RCC
5	31/F	cT1aN0	35	8	Papillary Clear Cell RCC
6	41/M	cT2N0	86	10	Clear Cell RCC
7	29/M	cT1bN0	40	8	Oncocytoma
8	30/F	cT1bN0	44	10	Papillary/Clear Cell RCC
9	32/F	cT1bN0	52	11	Clear Cell RCC
10	25/M	cT1bN0	45	11	Papillary/Clear Cell RCC Complicated cyst Oncocytoma/Fat poor AML
11	43/F	cT1bN0	50	9	Papillary RCC/Oncocytoma Fat poop AML
12	41/F	cT2N0	73	10	Oncocytoma
13	43/M	cT3aN0	33	7	Clear Cell RCC
14	38/F	cT1bN0	50	8	Bleeding Clear Cell RCC
15	32/M	cT3aN0	55	10	Clear Cell RCC
16	37/M	cT2N0	93	11	Clear Cell RCC

M = male; F = female; c = clinical; T = Tumor stage; N = Nodes involvement.

**Table 4:** Surgical approach, pathological stage, pathological diagnosis, and concordance with Computer Tomography diagnosis.

ID	Surgical Approach	Histological Stage	Histological Exam	Concordance Histological-CT Diagnosis
1	Retroperitoneoscopic radical nephrectomy	pT2N0	Clear Cell RCC Furhman 2	Yes
2	Open radical nephrectomy	pT1bN0	Clear Cell RCC Furhman 1	Yes
3	Retroperitoneoscopic radical nephrectomy	pT1bN0	Papillary-RCC, type II	Yes/No
4	Open Radical nephrectomy	pT2N0	Cystic Nephroma	No
5	Retroperitoneoscopic radical nephrectomy	pT1aN0	Clear Cell RCC Furhman 1	Yes/No
6	Open radical nephrectomy	pT2N0	Clear Cell RCCFurhman 2	Yes
7	Open partial nephrectomy with local renal hypothermia and hilar clamping	PT1bN0	Multicentric Chromophobe-RCC	No
8	Open partial nephrectomy with local renal hypothermia and hilar clamping	pT1bN0	Clear Cell RCC Furhman 1	Yes/No
9	Retroperitoneoscopic radical nephrectomy	pT1bN0	Clear Cell RCC Furhman 2	Yes
10	Open partial nephrectomy with local renal hypothermia and hilar clamping	pT1bN0	Multiloculated cystic Clear Cell RCC Furhman 1	No
11	Open partial nephrectomy with local renal hypothermia and hilar clamping	pT1bN0	Metanephric adenofibroma	No
12	Open radical nephrectomy	pT2N0	Oncocytoma	Yes
13	Open partial nephrectomy–segmentary renal vein invasion–radical nephrectomy	pT3aN0	Clear Cell RCC Furhman 1	Yes
14	Open partial nephrectomy with local renal hypothermia and hilar clamping	pT1bN0	Papillary-RCC, type I	No
15	Open radical nephrectomy	pT3aN0	Clear Cell RCC Furhman 1	Yes
16	Retroperitoneoscopic radical nephrectomy	pT2N0	Clear Cell RCC Furhman 1	Yes

RCC = renal cell carcinoma.
